# A new perspective on phylogeny and evolution of tetraodontiform fishes (Pisces: Acanthopterygii) based on whole mitochondrial genome sequences: Basal ecological diversification?

**DOI:** 10.1186/1471-2148-8-212

**Published:** 2008-07-19

**Authors:** Yusuke Yamanoue, Masaki Miya, Keiichi Matsuura, Masaya Katoh, Harumi Sakai, Mutsumi Nishida

**Affiliations:** 1 Ocean Research Institute, University of Tokyo, 1-15-1 Minamidai, Nakano-ku, Tokyo 164-8639, Japan; 2 Department of Aquatic Bioscience, Graduate School of Agricultural and Life Sciences, University of Tokyo, 1-1-1 Yayoi, Bunkyo-ku, Tokyo 113-8675, Japan; 3 Department of Zoology, Natural History Museum and Institute, Chiba, 955-2 Aoba-cho, Chuo-ku, Chiba 260-8682, Japan; 4 Collection Center, National Museum of Nature and Science, 3-23-1 Hyakunin-cho, Shinjuku-ku, Tokyo 169-0073, Japan; 5 Ishigaki Tropical Station, Seikai National Fisheries Research Institute, Fisheries Research Agency, 148-446 Fukai-Ohta, Ishigaki, Okinawa 907-0451, Japan; 6 Graduate School of Fisheries Science, National Fisheries University, 2-7-1 Nagata-Honmachi, Shimonoseki, Yamaguchi 759-6595, Japan

## Abstract

**Background:**

The order Tetraodontiformes consists of approximately 429 species of fishes in nine families. Members of the order exhibit striking morphological diversity and radiated into various habitats such as freshwater, brackish and coastal waters, open seas, and deep waters along continental shelves and slopes. Despite extensive studies based on both morphology and molecules, there has been no clear resolution except for monophyly of each family and sister-group relationships of Diodontidae + Tetraodontidae and Balistidae + Monacanthidae. To address phylogenetic questions of tetraodontiform fishes, we used whole mitochondrial genome (mitogenome) sequences from 27 selected species (data for 11 species were newly determined during this study) that fully represent all families and subfamilies of Tetraodontiformes (except for Hollardinae of the Triacanthodidae). Partitioned maximum likelihood (ML) and Bayesian analyses were performed on two data sets comprising concatenated nucleotide sequences from 13 protein-coding genes (all positions included; third codon positions converted into purine [R] and pyrimidine [Y]), 22 transfer RNA and two ribosomal RNA genes (total positions = 15,084).

**Results:**

The resultant tree topologies from the two data sets were congruent, with many internal branches showing high support values. The mitogenomic data strongly supported monophyly of all families and subfamilies (except the Tetraodontinae) and sister-group relationships of Balistidae + Monacanthidae and Tetraodontidae + Diodontidae, confirming the results of previous studies. However, we also found two unexpected basal splits into Tetraodontoidei (Triacanthidae + Balistidae + Monacanthidae + Tetraodontidae + Diodontidae + Molidae) and Triacanthodoidei (Ostraciidae + Triodontidae + Triacanthodidae).

**Conclusion:**

This basal split into the two clades has never been reported and challenges previously proposed hypotheses based on both morphology and nuclear gene sequences. It is likely that the basal split had involved ecological diversification, because most members of Tetraodontoidei exclusively occur in shallow waters (freshwater, brackish and coastal waters, and open seas), while those of Triacanthodoidei occur mainly in relatively deep waters along continental shelves and slopes except for more derived ostraciids. This suggests that the basal split between the two clades led to subsequent radiation into the two different habitats.

## Background

The order Tetraodontiformes comprises 429 species classified into 8–10 families [1-9]. As expected from the relatively large number of families for the indicated species diversity (8–10 vs. 429), members of the order are very morphologically diverse. For example, boxfishes have carapaces; tetraodontoids (except for *Triodon macropterus*) lack pelvic elements; and ocean sunfishes (Molidae) lack entire elements of the caudal fin. Tetraodontiforms also vary greatly in size: ocean sunfishes may grow up to 4 m in total length, while adult filefishes (*Rudarius minutus*) are less than 1 cm in standard length [10]. In addition, pufferfishes have compact genomes of approximately 400 Mb [11]. Much attention has been paid to two species of pufferfish, *Takifugu rubripes *[12] and *Tetraodon nigroviridis *[13], and the whole genome sequences of both have been published [14,15]. Many tetraodontiform fishes have been radiated into various habitats in temperate to tropical regions such as rocky and coral reefs, brackish and freshwaters, deep waters along continental shelves and slopes, and open oceans.

**Figure 1:**
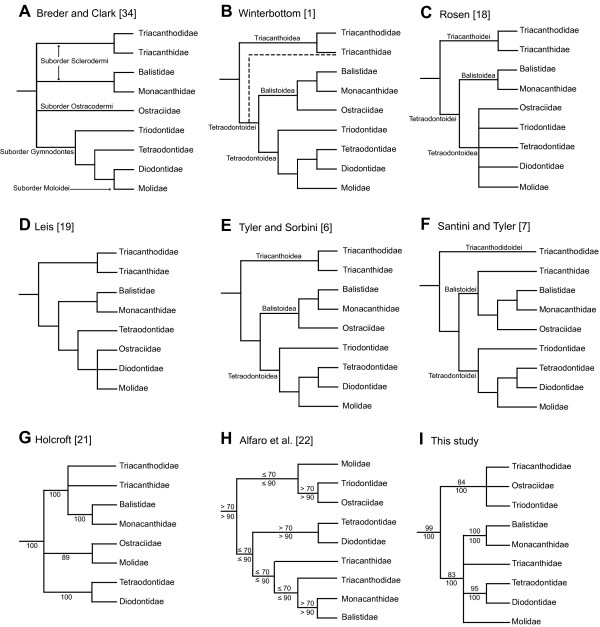
**Alternative phylogenetic hypotheses of the interfamilial relationships among Tetraodontiformes**. All family names follow Nelson [8]. Holcroft [21] and Leis [19] did not include the Triodontidae in their analyses. Numbers near branches indicate bootstrap values (above) and Bayesian posterior probabilities (below).

There are several hypotheses regarding the phylogeny of tetraodontiform families. Some of the families exhibit great reduction of skeletal elements, and many early studies generally divided the order into two groups, the Sclerodermi and Gymnodontes [4,16,17]. Scleroderms were considered to be primitive tetraodontiforms, usually having a set of primitive characters such as pelvic fin elements, separate teeth, and spinous dorsal fins. Gymnodonts were considered to be derived tetraodontiforms, usually having reductive characters such as no pelvic fin elements, teeth modified into a parrot-like beak, and no spinous dorsal fins. Traditionally, the Sclerodermi was further divided into two superfamilies (Triacanthoidea and Balistoidea), while the Gymnodontes was equal to the superfamily Tetraodontoidea. In most of phylogenetic studies, a series of the reduction was regarded to parsimoniously occur in derived lineages, and their phylogenetic relationships generally have been proposed to be (Triacanthoidea (Balistoidea, Tetraodontoidea)) (see Fig. 1).

Several authors have investigated the interrelationships of tetraodontiform fishes via cladistic analyses based on comparative osteology [6,7,18], ontogeny [19], myology [1], and karyology [20] and their results are similar to each other (Figs. 1A–F). Holcroft [21] and Alfaro et al. [22] determined the nuclear RAG1 gene and mitochondrial 12S and 16S rRNA gene sequences of representative tetraodontiform lineages and estimated their relationships (Figs. 1G and 1H). Both studies did not obtain clear resolution for basal relationships but only two sister-group relationships (Balistidae + Monacanthidae and Tetraodontidae + Diodontidae) with confidence. Therefore, many phylogenetic questions in the Tetraodontiformes, especially their basal relationships, remain unclear.

Whole mitogenome sequences from many teleost lineages have been determined and used for phylogenetic analyses with purposeful taxonomic sampling, which have successfully resolved many controversial issues in systematic ichthyology [23-29]. To address the questions regarding the phylogenetic relationships of the families and subfamilies of Tetraodontiformes, we purposefully chose 11 species in addition to the 14-tetraodontiform species used by Yamanoue et al. [30-32]. Together, these represent all families and subfamilies of the Tetraodontiformes, except for the Hollardinae. We determined whole mitogenome sequences for these 11 species, aligned them with the published sequences of the other 16 species, including two outgroups (total of 27 species), and conducted partitioned maximum likelihood (ML) and Bayesian phylogenetic analyses.

## Results

The complete L-strand nucleotide sequences from the mitogenomes of the 11 species (except for a portion of the putative control region for *Anoplocapros lenticularis*) were deposited in DDBJ/EMBL/GenBank (See Table 1). The genome content of the 11 species included two rRNA, 22 tRNA, and 13 protein-coding genes, plus the putative control region, as found in other vertebrates. Their gene arrangements were identical to the typical gene order of vertebrates.

**Table 1:** List of species analyzed, with DDBJ/EMBL/GenBank Accession numbers. Classification follow Nelson [8].

Classification	Species	Accession No.
Order Perciformes		
Family Caesionidae	*Pterocaesio tile*^2^	[DDBJ: AP004447]
Order Zeiformes		
Suborder Caproidei		
Family Caproidae	*Antigonia capros*^1^	[DDBJ: AP002943]
Order Tetraodontiformes		
Family Triacanthodidae	*Triacanthodes anomalus*^5^	[DDBJ: AP009172]
	*Macrorhamphosodes uradoi*^5^	[DDBJ: AP009171]
Family Triacanthidae	*Triacanthus biaculeatus*^5^	[DDBJ: AP009174]
	*Trixiphichthys weberi*^5^	[DDBJ: AP009173]
Family Balistidae	*Sufflamen fraenatum*^2^	[DDBJ: AP004456]
	Xenobalistes tumidipectoris*	[DDBJ: AP009182]
Family Monacanthidae	Aluterus scriptus*	[DDBJ: AP009183]
	Cantherhines pardalis*	[DDBJ: AP009184]
	*Stephanolepis cirrhifer*^1^	[DDBJ: AP002952]
	Thamnaconus modestus*	[DDBJ: AP009185]
Family Ostraciidae		
Subfamily Aracaninae	Anoplocapros lenticularis*	[DDBJ: AP009186]
	*Kentrocapros aculeatus*^5^	[DDBJ: AP009175]
Subfamily Ostraciinae	Lactoria diaphana*	[DDBJ: AP009187]
	*Ostracion immaculatus*^5^	[DDBJ: AP009176]
Family Triodontidae	*Triodon macropterus*^5^	[DDBJ: AP009170]
Family Tetraodontidae		
Subfamily Tetraodontinae	Arothron firmamentum*	[DDBJ: AP006742]
	*Takifugu rubripes*^4^	[DDBJ: AP006045]
	*Tetraodon nigroviridis*^4^	[DDBJ: AP006046]
	Sphoeroides pachygaster*	[DDBJ: AP006745]
Subfamily Canthigasterinae	Canthigaster coronata*	[DDBJ: AP006743]
	Canthigaster rivulata*	[DDBJ: AP006744]
Family Diodontidae	Chilomycterus reticulatus*	[DDBJ: AP009188]
	*Diodon holocanthus*^5^	[DDBJ: AP009177]
Family Molidae	*Mola mola*^3^	[DDBJ: AP006238]
	*Ranzania laevis*^1^	[DDBJ: AP006047]

*Newly determined in this study; ^1^Miya et al. [24]; ^2^Miya et al. [29]; ^3^Yamanoue et al. [32]; ^4^Yamanoue et al. [37]; ^5^Yamanoue et al. [40].

**Figure 2:**
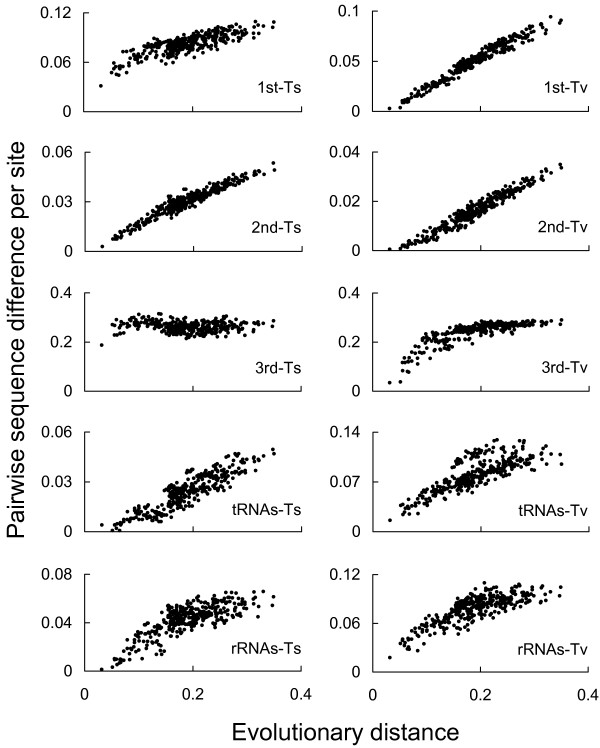
**Patterns of sequence variation in the mitochondrial genomes of 25 tetraodontiforms and two outgroups**. Pairwise transitional (TS) and transversional (TV) substitutions per site were plotted against evolutionary distance as a substitute for absolute geological time. Gamma-corrected maximum likelihood distances using the mtREV + F model [75] and derived from deduced amino acid sequences for the 13 protein-coding genes were used for evolutionary distances.

Both the pairwise transitional (TS) and transversional (TV) differences for each partition increased with increasing evolutionary distance, with the exception of the TS differences at the third codon position of protein-coding genes (Fig. 2), in which marked saturation has been observed in early stages of evolution (< 0.04 evolutionary distance) with no increases thereafter. It was apparent that some degree of saturation also occurred at other positions (particularly those in TSs), although pairwise differences seemed to accumulate steadily along the time axis.

**Figure 3:**
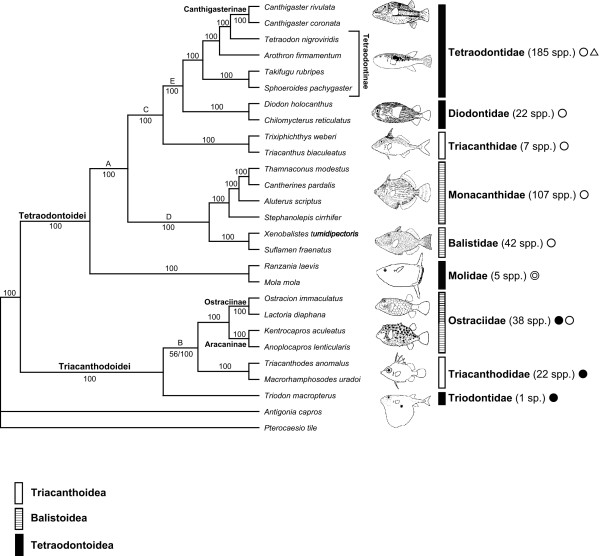
**Bayesian tree using the 12_n_3_r_RT_n _data set**. Bayesian analysis for the 123_n_RT_n _data set produced an identical topology. The numbers near internal branches indicate Bayesian posterior probabilities for the 12_n_3_r_RT_n _(left) and 123_n_RT_n _(right) data sets (values less than 50% not shown). Single numbers indicate that the 12_n_3_r_RT_n _and 123_n_RT_n _data sets resulted in identical values. Solid, open, and double circles, and triangles indicated that main habitats of a family are deep waters, coastal waters, open sea, and brackish and freshwater, respectively. Superfamilial classification follow Winterbottom [1] and Tyler and Sorbini [6].

**Figure 4:**
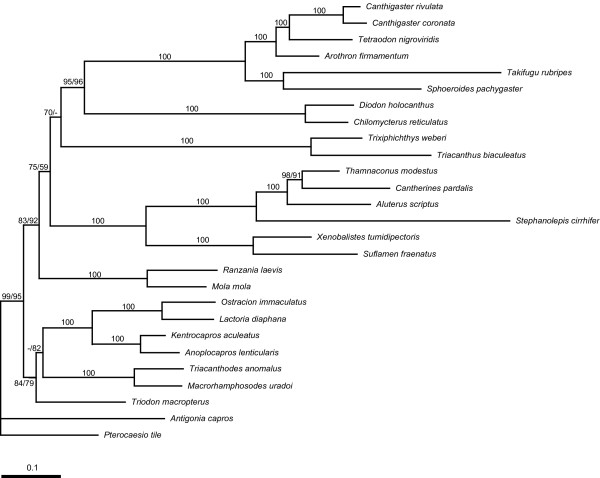
**Maximum likelihood (ML) tree with estimated branch lengths using the 12_n_3_r_RT_n _data set**. ML tree for the 12_n_3_r_RT_n _and 123_n_RT_n _data sets produced an identical topology with those of Bayesian tree. The numbers near internal branches indicate bootstrap probabilities for the 12_n_3_r_RT_n _(left) and 123_n_RT_n _(right) data sets (values less than 50% not shown). Single numbers indicate that the 12_n_3_r_RT_n _and 123_n_RT_n _data sets resulted in identical values. The scale indicates expected nucleotide substitution per site.

Although we were unable to determine a priori which data set recovered a more likely phylogeny, we considered that the 12_n_3_r_RT_n _data set (RY-coding) represented the best estimate of phylogenies, which effectively removes the likely noise from quickly saturated transitional changes in the third codon positions [27,33] and avoids a lack of signal by retaining all available positions in the data set [33]. Accordingly, the resultant tree from the 12_n_3_r_RT_n _data set derived from the partitioned ML and Bayesian analyses is shown in Figs. 3 and 4, with statistical support (bootstrap probabilities [BPs] from the partitioned ML analysis and posterior probabilities [PPs] from the partitioned Bayesian analysis) for 12_n_3_r_RT_n _and 123_n_RT_n _data sets indicated on each internal branch. No topological incongruities between the two data sets were found.

As in previous molecular analyses [21,22,32], our results indicated monophyly of the Tetraodontiformes (BPs = 95–99%; PPs = 100%), and supported monophyly of all tetraodontiform families and subfamilies with high statistical values (BPs and PPs = 100%) except for the paraphyletic subfamily Tetraodontinae (Fig. 3). Monophyly of the latter subfamily was rejected by statistical tests using the SH test (*p *= 0.012) and Bayes factor (Bayes Factor = 149.8). The mitogenomic data unambiguously supported sister-group relationships of Balistidae + Monacanthidae (Clade D: BPs and PPs = 100%) and Diodontidae + Tetraodontidae (Clade E: BPs = 95–96%; PPs = 100%), which have been reported in most previous morphological and molecular analyses (e.g., [1,6,7,19,21] shown in Figs. 1B–H) with a few exceptions [see Breder and Clark [34] (Fig. 1A), Shen and Wu [35]]. It should be noted that the two data sets consistently reproduced two unexpected clades herein designated as Tetraodontoidei and Triacanthodoidei with strong statistical support (BPs = 79–92%; PPs = 100%). However, this result may be affected by long-branch attraction because most of tetraodontoids and triacanthodoids comprise lineages with rapid and slow evolutionary rates of mitogenomes, respectively (Fig. 4).

## Discussion

### Phylogeny of tetraodontiform families

Ostraciidae have long been problematic because ostraciids exhibit mosaic morphologies (lacking pelvic fin elements and spinous dorsal fins, like tetraodontoids, but having separate teeth, like balistoids and triacanthoids). Some early studies classified the family into the Sclerodermi together with the Balistidae, Monacanthidae, and Triacanthoidea [16,17,36-39], although others placed the family in the monotypic Ostracodermi or Ostracoidea [5,34,40-43]. The sister-group relationship of Ostraciidae with Balistidae + Monacanthidae was also suggested by many previous authors [1,6,7,44,45], although Leis [19] and Rosen [18] placed the Ostraciidae within the tetraodontoids based on characters derived from early ontogeny and osteological characters, respectively (Figs. 1C and 1D). Subsequently, Britz and Johnson [46] reported unique characters shared solely by the Ostraciidae and Molidae (occipito-vertebral fusion), and argued that ostraciids should be included in the tetraodontoids. Our mitogenomic analyses placed the Ostraciidae within Triacanthodoidei, indicating close relationships with the Triacanthodidae and Triodontidae. Our statistical comparisons rejected all previous hypotheses of close relationships with Balistidae + Monacanthidae (*p *= 0.007, Bayes factor = 92.5), Molidae (*p *= 0.019, Bayes factor = 82.22), Diodontidae and Molidae (*p *< 0.001, Bayes factor = 145.58), and Triodontidae + Tetraodontidae + Diodontidae + Molidae (= Winterbottom's [1] Tetraodontoidea) (*p *= 0.014, Bayes factor = 110.34). Our results also rejected that of Leis [19] (*p *< 0.001; Bayes factor = 203.32) and Rosen [18] (*p *= 0.009; Bayes factor = 128.04).

Our placements of Triodontidae and Triacanthidae also differ from most of the previous hypotheses (Fig. 1). *Triodon macropterus*, a monotypic triodontid with a mosaic of primitive and derived morphological characters (ref, [4]; e.g., parrot-like beak and lacking pelvic fins, like tetraodontoids; spinous dorsal fins, a pelvis, and procurrent rays, like balistoids and triacanthoids). In addition, triodontids possess ribs, which all other tetraodontiform fishes possess except one species of filefishes (*Pseudaltarius nasicornis*). Although the Triodontidae have been considered the basalmost members of the Tetraodontoidea in most studies [1,4,6,7], this family has also occasionally been classified as a member of the Sclerodermi [17,39]. In our study, a close affinity of Triodontidae with Tetraodontidae + Diodontidae + Molidae was not marginally rejected by the SH test (*p *= 0.057) but was strongly rejected by the Bayes factor (110.34). The ML and Bayesian analyses of the mitogenomic data sets, however, strongly suggested that the Triodontidae belonged in Triacanthodoidei together with the Triacanthodidae and Ostraciidae (BPs and PPs = 100%).

Members of the Triacanthidae have primitive morphological characters including paired pelvic fin spines, spinous dorsal fins, and separate teeth, and are often treated as basal members together with the Triacanthodidae [1,4,6,18,19,34,47,48]. However, the cladistic analysis of myological characters by Winterbottom [1] implied a close relationship between triacanthids and not only triacanthodids but also higher tetraodontiforms (= Tetraodontoidei; Fig. 1B). The cladistic analysis of 219 morphological characters by Santini and Tyler [7] also suggested that triacanthids were the sister-group of Balistidae + Monacanthidae + Ostraciidae (Fig. 1F). Our results placed the Triacanthidae within Tetraodontoidei together with the Balistidae, Monacanthidae, Tetraodontidae, Diodontidae, and Molidae, but we did not obtain clear relationships within Tetraodontoidei. Its sister-group relationships with the following groups were not rejected by the SH test but were rejected by the Bayes factor: Triacanthodidae (*p *= 0.539, Bayes factor = 55.42) and Winterbottom's [1] Tetraodontoidei (*p *= 0.110, Bayes factor = 94.26).

### Comparison to previous molecular studies

Holcroft [21] reported the first molecular phylogenetic analysis of the tetraodontiform families (Fig. 1G). The study was based on the RAG1 gene and the mitochondrial 12S and 16S rRNA gene sequences, and Bayesian and maximum parsimony analyses using the RAG1 gene yielded a tree topology similar to previous morphological hypotheses in that the group once called Sclerodermi (i.e., Triacanthodidae, Triacanthidae, Balistidae, and Monacanthidae) was confirmed. In contrast, the tree topologies derived from the 12S and 16S rRNA genes (Figs. 6 and 7 in Holcroft [21]) were unorthodox, and she concluded that problematic alignment of the mitochondrial data could account for those results. Alfaro et al. [22] also employed partitioned ML and Bayesian analyses using concatenated sequences of the RAG1 and mitochondrial 12S and 16S genes from representatives of all families (Fig. 1H). Holcroft [21] and Alfaro et al. [22] similarly recovered the sister-group relationships of Tetraodontidae + Diodontidae and Balistidae + Monacanthidae, and the paraphyly of Tetraodontinae as in our resultant tree (Fig. 1I).

Our mitogenomic tree topology, however, is also incongruent with those derived from molecular analyses of the nuclear RAG1 genes by Holcroft [21] (Fig. 1G) as well as the RAG1/mitochondrial 12S and 16S rRNA genes by Alfaro et al. [22] (Fig. 1H). Holcroft [21] considered the tree derived from the RAG1 gene to be more reliable than those from the 12S and 16S rRNA genes, because there were ambiguities in alignment, apparent saturation in nucleotide substitutions along the time axis, and noticeable codon biases in the latter (see Holcroft [21]). Alfaro et al. [22] also presented tetraodontiform phylogeny using concatenated sequences of RAG1/12S and 16S rRNA genes with sequences of *Triodon macropterus *and another representative of the Triacanthidae added to the data set of Holcroft [21]. However, both studies failed to obtain clear resolution for the basal relationships of Tetraodontiformes (Figs. 1G and 1H), with a basal polytomy in Holcroft [21] and crown node with weak support values (BPs ≤ 70%, PPs ≤ 90%) in Alfaro et al. [22].

We conducted ML and Bayesian analyses using the data sets of Holcroft [21] (RAG1 only) and Alfaro et al. [22] with topological constraints on our phylogeny derived from the 12_n_3_r_RT_n _data set (Figs. 3 and 4). Statistical comparisons using the likelihood-based SH test and Bayes factor were conducted between the unconstrained and constrained trees based on the data sets of Holcroft [21] and Alfaro et al. [22]. The SH test based on the data set of Holcroft [21] did not reject our hypothesis (*p *= 0.067), but the Bayes factor very strongly rejected our hypothesis (Bayes factor = 37.6). Both the SH test and Bayes factor based on the data set of Alfaro et al. [22] rejected our hypothesis (*p *= 0.037 Bayes factor = 74.92), but the difference using the SH test was only marginally significant. Based on our mitogenome data set, however, the SH test and Bayes factor confidently rejected the topologies of both Holcroft [21] (*p *< 0.001; Bayes factor = 112.64) and Alfaro et al. [22] (*p *= 0.004; Bayes factor = 97.45).

Considering that the conservative SH test based on the data sets of Holcroft [21] and Alfaro et al. [22] showed no or only marginally significant differences among trees of RAG1 (*p *< 0.001) or RAG1/12S and 16S rRNA genes (*p *= 0.037) and mitogenomes, it seems likely that their data sets did not comprise suitable gene sequences with adequate taxonomic sampling for recovering a precise phylogeny of the Tetraodontiformes. Our taxonomic sampling represented all families by at least two species (except for monotypic Triodontidae) to avoid long-branch attraction. In contrast, the taxonomic sampling of Holcroft [21] did not include any triodontids and only one species each of Triacanthidae and Triacanthodidae, which would lead to long-branch attraction in the resulting tree [49-51]. Alfaro et al. [22] improved the data set by adding *Triodon macropterus *(Triodontidae) and *Pseudotriacanthus strigilifer *(Triacanthidae), but a single species was used for the Triacanthodidae. It may be argued that more noise-free (unsaturated) nuclear genes are superior for precisely estimating phylogenies [21,52,53]. However, Saitoh et al. [33] demonstrated that non-saturated partitions of the mitochondrial genes (second codon positions) along the time axis do not necessarily result in correct phylogenies among basal groups, due to a lack of phylogenetic signal in the data set; slowly evolving nuclear genes may lack phylogenetic signal.

Our mitogenomic data sets (15,084 bp) were much longer, and therefore should have far more phylogenetic signals and noise, than those based on the RAG1 gene (ca. 1400 bp) in Holcroft [21] and RAG1/12S and 16S rRNA genes (ca. 2500 bp) in Alfaro et al. [22]. While it is likely that randomly accumulated noise could be masked by phylogenetic signal, noise accumulated systematically may eventually lead to erroneous estimations of phylogenies. Thus, increasing the length of sequences with no or little systematic noise should be advantageous for estimating correct phylogenies, while increasing the length of sequences with significant systematic noise would lead to less accurate phylogenies. Mitochondrial genes encode basic functions, such as aerobic respiration, in mitochondria [54] and thus are likely to accumulate less systematic noise than functionally specialized nuclear genes. Moreover, our data sets were analyzed under appropriate substitution models, partitions, and various treatments of the third codon position (123_n_RT_n _and 12_n_3_r_RT_n_) to reduce as much phylogenetic noise as possible. Furthermore, the use of shorter sequences or a single gene generates phylogenetic hypotheses that are incongruent or lacking support [55-58], and increasing sequence length may be a better way to improve support, resolution, and accuracy of a difficult phylogeny (> 5000 bp in Hillis [59]; > 10,000 bp in Wortley et al. [60]). On the other hand, the possibility cannot ruled out that our result was erroneously estimated due to long branch attraction because the two unexpected clades have considerable differences in evolutionary rates of mitogenomes (Fig. 4). Accordingly, we cannot conclude that our mitogenomic analyses correctly estimated tetraodontiform phylogeny, and further taxonomic sampling and additional gene sequences would be needed to clarify these relationships.

### Ecological diversification

Tetraodontiform fishes are found primarily in coastal shallow waters and estuaries of tropical and temperate regions such as coral and rocky reefs, sandy and muddy bottoms, and sea weed beds [4,8,9,47,61]. Moreover, some pufferfishes are radiated into brackish and freshwater in Southeast Asia, Central Africa, and South American basins [62], and ocean sunfishes (Molidae), a few of tetraodontids (e.g., some species of *Lagocephalus *and *Sphoeroides*), and balistids (e.g., *Canthidermis maculata*) are widely distributed in open seas [8,9]. On the other hand, a few groups inhabit relatively deep waters of tropical and temperate regions. Triacanthodids are distributed in continental shelves and slopes in Indo-Pacific and Caribbean Sea [8,9,47]. Triodontids are also distributed in deep waters such as margins of continental shelves and slopes in tropical Indo-West Pacific [8,9]. Ostraciin ostraciids are generally found in shallow waters such as coral reef and near shores, but most aracanin ostraciids occur in continental shelves of temperate Indo-West Pacific [8,9]. A few tetraodontids (e.g., some species of *Lagocephalus *and *Sphoeroides*) and monacanthids (e.g., some species of *Thamnaconus*) are also found in deep waters such as continental shelves [9]. Our most striking finding was the two unexpected clades of Tetraodontiformes (Tetraodontoidei and Triacanthodoidei), which were strongly supported. The basal split of the Tetraodontiformes was implied by the mitogenomic analyses of Yamanoue et al. [32]. We found that the basal split is more congruent with the ecological diversification within the order than that expected from the traditional taxonomy based on morphology. Most members of Tetraodontoidei exclusively radiated into shallow waters (freshwater, brackish and coastal waters, and open seas), while those of Triacanthodoidei except for more derived ostraciids inhabit relatively deep waters along continental shelves and slopes [8,9]. This suggests that the basal split between the two clades led to subsequent radiation into the two different habitats. As mentioned above, a few species of Tetraodontoidei (tetraodontids and monacanthids) are found in relatively deep waters. However, it is probable that the center of dispersal for each group of Tetraodontoidei is apparently not deep waters but shallow waters because most members of tetraodontids and monacanthids, even those of the same genera with deep-sea inhabitants, are found in shallow waters. On the other hand, most members of Triacanthodoidei inhabit deep sea such as continental shelves and slopes. However, ostraciin ostraciids inhabit reefs and near shore [9]. Considering that most aracanin ostraciids, the plesiomorph sister-group of ostraciin ostraciids [1,4], occurred in relatively deep waters [8,9], it seems likely that ostraciin ostraciids were secondarily radiated into shallow waters. Alfaro et al. [22] proposed that reef inhabitants exhibit higher species diversity than non-reef species, and this hypothesis may help explain the difference in diversity between Tetraodontoidei (368 species) and Triacanthodoidei (61 species) observed in our study (Fig. 3).

Some molecular studies have reported a basal split according to habitat for some vertebrate groups in their early stage of evolution. Placental mammals were divided into the Afrotheria and the remaining groups, the former of which were initially restricted to Africa [63-65]. In addition, cichlids in the Great Lakes of East Africa are categorized according to which lake they inhabit (Victoria, Tanganyika, or Malawi [66-68]). Due to morphological convergence, the differences in these groups were previously undetectable until the emergence of molecular phylogenies. Accordingly, as is the case with our results, clear divergences in habitat at early stages of evolution were often overlooked.

## Conclusion

The phylogenetic analyses of whole mitogenomic data sets confirmed monophyly of all families and subfamilies (except the Tetraodontinae) and sister-group relationships of Balistidae + Monacanthidae and Tetraodontidae + Diodontidae as in the previous studies. We also found an unexpected basal splits into Tetraodontoidei (Triacanthidae + Balistidae + Monacanthidae + Tetraodontidae + Diodontidae + Molidae) and Triacanthodoidei (Ostraciidae + Triodontidae + Triacanthodidae), which has never been reported and challenges previously proposed hypotheses based on both morphology and nuclear sequences. The mitogenomic hypothesis seems more congruent with the basal ecological diversification within the order, because most members of Tetraodontoidei exclusively occur in shallow waters (freshwater, brackish and coastal waters, and open seas), while those of Triacanthodoidei occur in relatively deep waters along continental shelves and slopes except for more derived ostraciids. This suggests that the basal split between the two clades led to subsequent radiation into the two different habitats.

## Methods

### Taxonomic sampling

Our purposeful taxonomic sampling strategy was based on Hillis [49], who recommended selecting taxa within the monophyletic group of interest that will represent the overall diversity of the group and that are expected (based on current taxonomy or previous phylogenetic studies) to subdivide long branches in the initial tree (p5 in Hillis [49]). We chose at least two species from each family or subfamily, except for one species from the monotypic family Triodontidae and four species each from the speciose groups Tetraodontinae and Monacanthidae (See Table 1). No specimen of the subfamily Hollardinae of the Triacanthodidae was available for use in the present study. Final rooting was done using the borefish *Antigonia capros *and the dark-banded fusilier *Pterocaesio tile*, based on the results of Yamanoue et al. [32]. Table 1 lists all species used in this study, with their DDBJ/EMBL/GenBank accession numbers.

#### DNA extraction, PCR, and sequencing

A portion of the epaxial musculature (ca. 0.25 g) was excised from fresh specimens of each species and immediately preserved in 99.5% ethanol. Total genomic DNA was extracted using a Qiagen DNeasy tissue kit (Qiagen) following the manufacturer's protocol. The mitogenomes were amplified in their entirety using a long PCR technique [69]. Four fish-versatile long PCR primers were used in various combinations to amplify the entire mitogenome in two reactions. The long PCR products were diluted with TE buffer (1:19) for subsequent uses as PCR templates.

A total of 148 fish-versatile PCR primers were used in various combinations to amplify the contiguous, overlapping segments of the entire mitogenome, and 11 species-specific primers were designed when no appropriate primers were available. A list of the PCR primers used in this study is available from Y.Y. upon request. Long PCR and subsequent short PCR were performed as previously described [26,70].

Double-stranded PCR products, purified using a ExoSAP-IT (USB), were subsequently used for direct cycle sequencing with dye-labeled terminators (Applied Biosystems). The primers used were the same as those for PCR. All sequencing reactions were performed according to the manufacturer's instructions. Labeled fragments were analyzed using Model 377 and 3100 DNA sequencers (Applied Biosystems).

### Alignment

The DNA sequences were edited and analyzed with EDITVIEW (version 1.0.1), AUTOASSEMBLER (version 2.1) (Applied Biosystems), and DNASIS (version 3.2) (Hitachi Software Engineering). A total of 13 protein-coding, 22 tRNA, and two rRNA gene sequences for 27 species were aligned using PROALIGN (version 0.5) [71]. All sequences from L-strand-encoded genes (ND6 and eight tRNA genes) were converted to complementary strand sequences. Amino acids were used for alignments of the protein-coding genes. Regions with posterior probabilities of = 70% were used in the phylogenetic analyses. Unambiguously aligned sequences were 11,340, 1494, and 2250 nucleotide positions from the 13 protein-coding genes, 22 tRNA genes, and two rRNA genes, respectively (total of 15,084 positions).

We constructed two different data sets to see the effects of quickly saturating the third codon positions in the protein-coding genes on the estimation of phylogeny: 1) all aligned positions of gene-coding regions of mitogenomic sequences (designated as 123_n_RT_n_, where n denotes nucleotides; total of 15,084 positions) and 2) the third codon positions converted to purine (R) and pyrimidine (Y) (12_n_3_r_RT_n_, where r denotes RY-coding [72,73]). The aligned sequence data in NEXUS format are available from Y.Y. upon request.

### Analysis of sequence variations

Pairwise comparisons and statistical information from the mitogenomic sequences were obtained using PAUP (version 4.0b10) [74]. To examine patterns of sequence variation in the first, second, and third codon positions, and separately for the protein-coding genes, rRNA, and tRNA, we plotted pairwise nucleotide differences (sorted into transitional [TS] and transversional [TV] differences) against evolutionary distance as a substitute for absolute geological time. The gamma-corrected maximum-likelihood (ML) distance with the mtREV + F model [75] derived from concatenated amino acid sequences from the 13 protein-coding genes was calculated with TREE-PUZZLE (version 5.2) [76] and used as the evolutionary distance. The resulting distances of this method have been demonstrated to be linear with absolute geological time for several vertebrate taxa [77].

### Phylogenetic analysis

Maximum likelihood (ML) analysis has traditionally not been feasible with a large data set such as the one used in this study. However, a recently developed program, RAXML [78], has greatly improved ML analysis by implementing a novel, rapid-hill-climbing algorithm. This program performs heuristic phylogenetic searches under general time -reversible (GTR) model sites following a discrete gamma distribution (ref. [79]; GTR + Γ) and also allows data partitioning. This program produces likelihood values using GTRCAT, which is a GTR approximation with optimization of individual per-site substitution rates and classification of those individual rates into a certain number of rate categories. GTRCAT allows the integration of rate heterogeneity into phylogenetic analyses at significantly lower computational and memory costs; however, the approximation is numerically instable. To reconstruct the ML tree, we selected GTRMIX as a nucleotide -substitution model, which makes RAXML perform a tree inference (search for a good topology) under the GTRCAT model. In the GTRMIX model, when the analysis is finished, RAXML switches to GTRGAMMA to evaluate the final tree topology to yield stable likelihood values.

We set five (123_n_RT_n _and 12_n_3_r_RT_n_) partitions, assuming that functional constraints on sequence evolution are more similar within codon positions (or types of molecules) across genes than across codon positions (or types of molecules) within genes, at least for a set of mitochondrial genes. We performed 100 inferences in each analysis and found the best ML tree by comparing final likelihoods among them. To evaluate the robustness of the internal branches of the ML tree, 100 bootstrap replications were calculated for each data set without data partitioning (data partitioning is not feasible for bootstrapping in the current version of RAXML). The GTRCAT model was used for bootstrap analyses because we were only interested in the bootstrapped topologies.

Partitioned Bayesian phylogenetic analyses were conducted with MRBAYES (version 3.1.2) [80], which allows up to 150 partitions and the use of complex substitution models with independent parameters for each partition. The GTR model, with some sites assumed to be invariable and with variable sites assumed to follow a discrete gamma distribution (ref. [79]; GTR + I + Γ), was selected as the best-fit model of the nucleotide substitution for each partition on the basis of the Akaike Information Criteria (ref. [81]; AIC). The best-fit model was selected using MRMODELTEST (version 2.1) [82], which is a simplified version of MODELTEST (version 3.06) [83]. We set the GTR + I + Γ model of nucleotide substitutions in MrBayes as follows: "lset nst = 6" (GTR) and "rates = invgamma" (I + Γ). We assumed that all model parameters were unlinked and the rate multipliers were variable across partitions, which were set in MrBayes as follows: "unlink revmat = (all) pinvar = (all) shape = (all) statefreq = (all)" (unlinking substitution rates of the GTR model, proportion of invariable sites, gamma shape parameters, and base frequency across all partitions) and "prset ratepr = variable" (rate multipliers variable across partitions). We used the default settings for the priors on the proportion of invariable site (0–1) and gamma shape parameters (0.1–50.0). A Dirichlet distribution was assumed for the rate matrix and base frequency, and every tree topology was assumed to be equally probable.

The Markov chain Monte Carlo (MCMC) process was set so that two independent analyses starting from different random trees (nruns = 2) with four chains (three heated and one cold) ran simultaneously. On the basis of two to four preliminary runs with varying cycles (1.0–3.0 × 10^6^), we estimated average log likelihood scores at stationarity (123_n_RT_n _≈ -165,180; 12_n_3_r_RT_n _≈ -106,860), and subsequently conducted two independent runs for each data set. After reaching stationarity in the two runs, we continued both runs for 1.0 × 10^6 ^cycles with one in every 100 trees being sampled (10,000 trees) for all data sets. Thus, we determined the posterior probabilities of the phylogenies and its branches based on 20,000 trees pooled from the two runs for the two data sets.

### Testing alternative hypotheses

Alternative tree topologies were individually compared to the resulting ML tree using the likelihood-based SH test [84] implemented in PAUP [74]. We conducted ML analyses using RAXML with constrained topology and estimated ML trees with those constraints using a GTR + I + Γ model of sequence evolution, implemented in PAUP. Then we estimated the variance in likelihood difference between two topologies using the resampling estimated log-likelihood (RELL) method from 1000 bootstrap replications, and the difference was statistically evaluated. A value of *p *< 0.05 was considered significantly different. The data set excluding *Triodon macropterus *was reconstructed for statistical comparisons with Holcroft [21] and Leis [19], who did not use triodontids in their analyses.

We also tested alternative tree topologies with a monophyletic subgroup as the null hypothesis with Bayes factors, using the constraint option in MrBayes, and analyzed each data set as previously described. We calculated the harmonic means of likelihoods after the burn-in period using the sump command in MrBayes, and likelihood values were compared to those values from the unconstrained analyses by calculating twice the differences (i.e., 2 *Δln). Following Kass and Raftery [85], a 2*Δln Bayes factor of > 10 was interpreted as strong evidence for rejecting the null hypothesis. It should be noted, however, that Brandley et al. [86] found that a 2*Δln Bayes factor of 10 could be a less conservative threshold.

## Authors' contributions

YY, MM, KM, and MN designed the study, and all authors were involved in sampling. YY carried out molecular work, analyzed the data, and drafted the manuscript. MM, KM, MK, HS, and MN helped draft the manuscript. All authors read and approved the final manuscript.
